# Chironomids (Insecta, Diptera, Chironomidae) from alpine lakes in the Eastern Carpathians with comments on newly-recorded species from Ukraine

**DOI:** 10.3897/BDJ.8.e49378

**Published:** 2020-05-19

**Authors:** Peter Bitušík, Milan Novikmec, Ladislav Hamerlik

**Affiliations:** 1 Faculty of Natural Sciences, Matej Bel University, Banská Bystrica, Slovakia Faculty of Natural Sciences, Matej Bel University Banská Bystrica Slovakia; 2 Faculty of Ecology and Environmental Sciences, Technical University in Zvolen, Zvolen, Slovakia Faculty of Ecology and Environmental Sciences, Technical University in Zvolen Zvolen Slovakia

**Keywords:** Non-biting midges, alpine ponds, pupal exuviae, new records, Ukrainian Carpathians

## Abstract

**Background:**

The first summarising checklist of Ukrainian Chironomidae (Insecta, Diptera) consisted of 302 species. Compared to other European countries, it is obvious that the real chironomid diversity of Ukraine has not been fully documented and greater effort is needed to discover the actual richness of this family. Thus, our survey focused on the chironomid fauna of some alpine lakes situated above the treeline in the Ukrainian Carpathians (a part of the Eastern Carpathians) aiming to contribute to the knowledge of the Ukrainian chironomid fauna and create the basis for more comprehensive neo- and palaeolimnological studies of these, regionally, little-known ecosystems.

**New information:**

In total, 34 species/taxa, belonging to 22 genera and 4 subfamilies were collected in June 2019. Ten species were recorded for the first time in Ukraine: *Zavrelimyia melanura*, *Acamptocladius reissi, Cricotopusspeciosus, Cricotopuscurtus, Heterotrissocladius marcidus*, *Orthocladius dentifer*, *Psectrocladiusoligosetus, Polypedilumuncinatum, Paratanytarsus laccophilus* and *Tanytarsus bathophilus*. The occurrence of six species previously considered as “doubtfully present” in Ukraine was finally confirmed. Generally, the surveyed lakes have a unique composition of chironomids consisting of a mixture of species typical for cold alpine lakes and acidic ponds situated at lower altitudes.

## Introduction

The Chironomidae family is a group of holometabolous insects distributed with the widest range of any family of insects, with individual species occurring from Antarctica and sub-Antarctic islands to Ellesmere Island in the Canadian Arctic. In this respect, chironomids are exceeded only by a few collembolan and mite species (Cranston 1995). Chironomids are common inhabitants of most aquatic habitats and regularly dominate aquatic insect communities in both abundance and species richness, often approaching 80 or more species and occasionally exceeding 100 species per site (Ferrington 2007).

Due to their ecological diversity, ubiquity and critical position in food webs, chironomids have been important components of biomonitoring and conservation programmes (see Nicacio and Juen 2015 for review). In addition to being important for understanding contemporary, mainly anthropogenic impacts, chironomid subfossil remains represent a tool for reconstructing past environmental changes (e.g. Langdon et al. 2010).

From the estimated more than 10,000 species worldwide (Cranston 1995), nearly 1300 species have been recorded in Europe (Spies and Sæther 2013). Naturally, there are considerable differences in knowledge of regional chironomid faunas, with Western Europe having the most comprehensive knowledge as a result of higher concentration of specialists and longer history of the chironomid investigation relative to other regions (e.g. Ferrington 2007.

According to the first summarising checklist, 302 Chironomidae species have been recorded from Ukraine (Baranov 2011a). However, as the author of that publication stated, most of the data are probably based on identifications of larval stages only and, thus, often not reliable. The expansion of knowledge on Ukrainian chironomids has continued in the last decade, evidenced by the discovery of new regional records and even several new species (Lietytska and Baranov 2009, Baranov 2011b, Baranov 2013, Baranov 2015, Baranov and Ferrington Jr. 2013, Baranov and Przhiboro 2014, Moubayed-Breil and Baranov 2018 and citations therein).

Here, we provide the first inventory of the family Chironomidae from some alpine lakes of the Eastern Carpathians located in Ukraine. Out of the several thousand natural and artificial lakes in Ukraine (Polishchuk and Igumnova 1983), mountain lakes located in the Carpathians represent a tiny fraction in both number and size. Nevertheless, these alpine lakes are particularly suitable for studying ecosystem responses to environmental impacts both global (e.g. climate change, atmospheric pollution) and regional (e.g. land use change, species introductions; Catalan and Donato Rondón 2016). Our survey not only expands the knowledge on the Ukrainian chironomid fauna, but can also serve as the first step to more comprehensive neo- and palaeolimnological studies of these unique and, in Ukraine, so far largely unknown ecosystems.

## Materials and methods

### Study area and sampling sites

**Figure 1.**
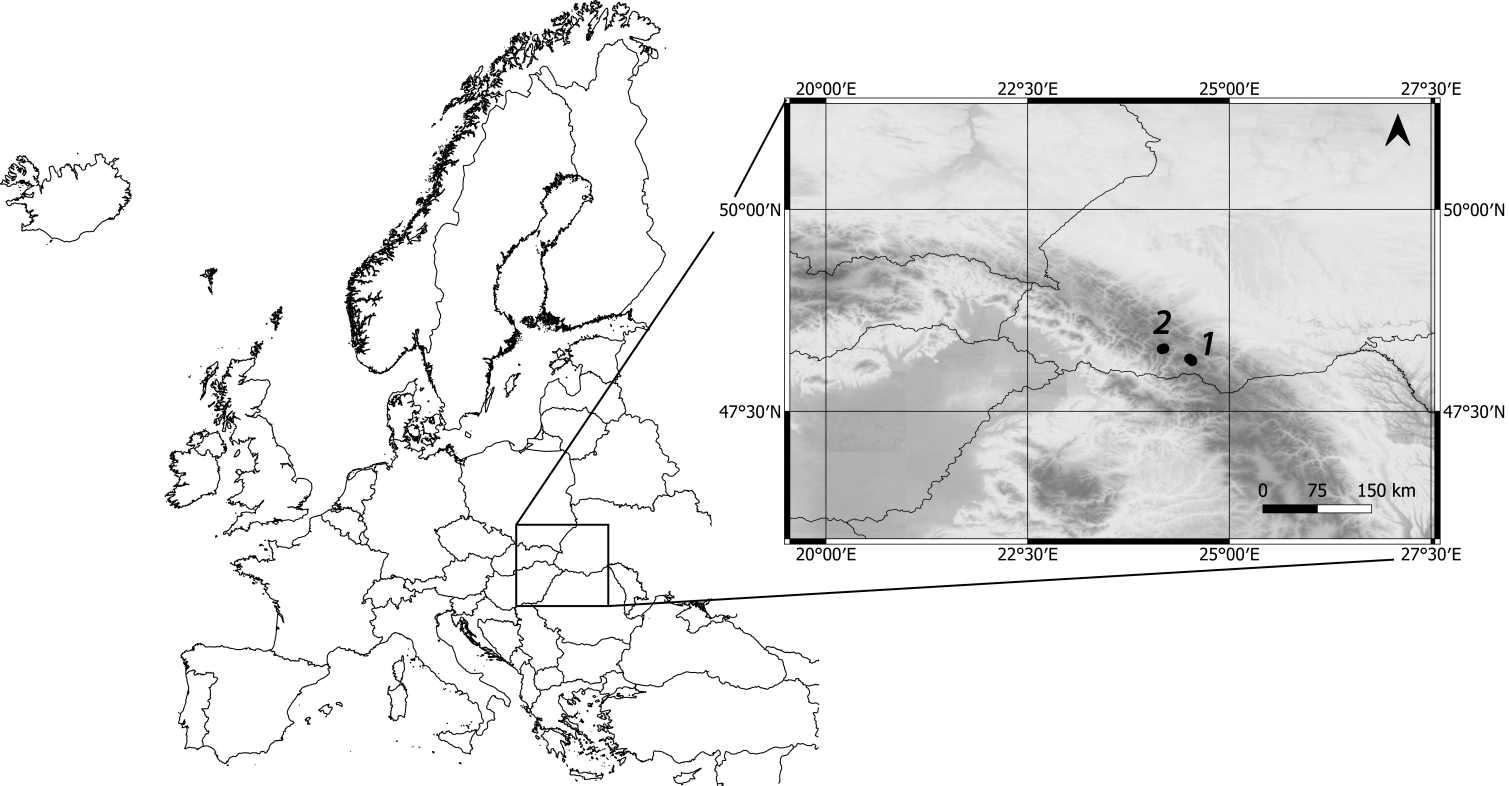
Geographical location of the studied area and and the position of sampling sites in the Eastern Carpathians (1 – Chornohora lakes: Breskul 1, Breskul 2, Nesamovyte, Dantsyzh; 2 – Svydovets lakes: Geryshaska, Vorozheska 1, Vorozheska 2, Vorozheska 3).

**Figure 2.**
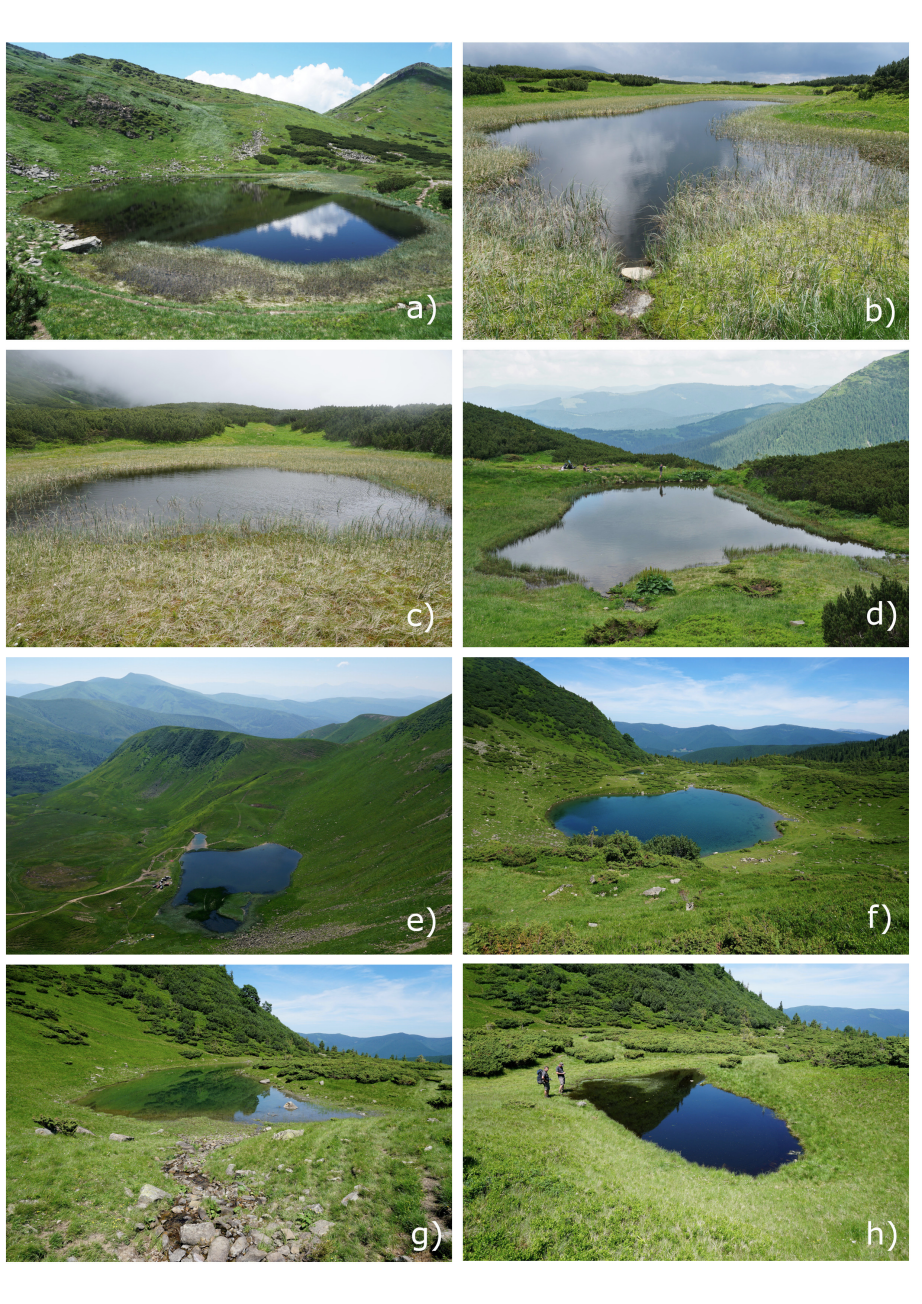
View of the study lakes. **a.** Nesamovyte; **b.** Breskul 1; **c.** Breskul 2; **d.** Dantsyzh; **e.** Geryshaska; **f.** Vorozheska 1; **g.** Vorozheska 2; **h.** Vorozheska 3. For basic characteristics and coordinates, see Table 1.

The part of the Eastern Carpathians located in Ukraine (Ukrainian Carpathians) represents medium altitude mountains with only few peaks slightly exceeding 2000 m a.s.l. The highest massifs, Chornohora and Svydovets, show direct glacial imprints of past glaciations (Matoshko 2011). The glacial cirques and glacial valleys, usually separated by a rock step, are the most remarkable signs of glacier activity. In some cirques, lakes of glacial origin formed, although most of them are in advanced terrestrial phase or have turned to peat bogs. The present study was performed at eight lakes in Chornohora and Svydovets Massifs (Fig. 1, Fig. 2) between 23^th^ and 26^th^ June 2019. In case we were not aware of official lake names, we named the lakes for adjacent hills (Breskul 1, Breskul 2, Dantsyzh) or nearby named lakes (Vorozheska 2, Vorozheska 3). Due to their small size (< 2 ha, max depth < 2.2 m) and high elevation (above the upper tree line), all the study sites can be considered alpine ponds (Céréghino et al. 2007, Hamerlík et al. 2013).

In the study area, bedrock is represented by sedimentary rocks of Cretaceous-Paleogene flysch. The dominant vegetation of the lake catchment areas is formed by unique mountain grasslands (“polonyna”) chequered, to various extents, by juniper (*Juniperus communis nana* (Willd.) Syme), dwarf pine (*Pinus mugo* Turra) or rhododendrons (*Rhododendron kotschyi* Simonkai) patches at some lakes. The studied lakes are located at altitudes between 1477 and 1745 m. The bottoms of the lakes vary from stony silt to mud and organic depositions.

Coordinates of studied lakes were identified in the field using GPS device Garmin GPSmap 64. Lake area was estimated in Google Earth Pro. Maximum lake depth was estimated in the field, except for one site, where published data were available. Basic characteristics of the studied lakes are presented in Table 1.

### Sampling methods

Floating chironomid pupal exuviae and drowned adults were collected along the shores of lakes at stretches by skimming the water surface with a hand net (mesh size 250 μm, frame diameter 25 cm) with a telescopic handle. The collected material was placed into labelled plastic bottles and preserved with 75% ethanol. Sorted exuviae and adult males were mounted on microscopic slides and identified using Schlee (1968), Langton and Visser (2003), Ekrem (2008), Stur and Ekrem (2006), Langton et al. (2013) for pupal exuviae and Langton and Pinder (2007a), Langton and Pinder (2007b) for adults. The nomenclature and distribution of species follow Fauna Europaea (Spies and Sæther 2013). Voucher specimens are deposited in the collections of the Dept. of Biology and Ecology, Faculty of Natural Sciences, Matej Bel University in Banská Bystrica.

## Data resources

A total of 1,124 pupal exuviae, 7 pharate adults (males) and 35 adults (males) were identified to 22 genera, 34 species/ taxa and 4 subfamilies: Orthocladiinae were represented with 15 species/ taxa, followed by Chironominae (15), Tanypodinae (3) and Prodiamesinae (1) (Table 2).

The number of species/taxa in a single lake varied from 4 to 13 with the mean diversity being 8.5 taxa per lake. The most frequent taxa were *Chironomus* spp. (6 lakes) followed by *Synendotendipes* sp. (most likely *S. dispar*, as the collected adults, 5 lakes); Cladotanytarsus (s. str.) atridorsum and *Paratanytarsus laccophilus* were recorded in half of the lakes. Half of the species (17) were recorded in a single lake only.

In some cases, pupal exuviae characteristics allowed identification to morphotypes only that may not correspond to valid species: *Corynoneura* Pe 2a, Cricotopus (Isocladius) Pe 5, *Chironomus* lob-pe 2a, genus *Synendotendipes*, *Tanytarsus* Pe 4c. Due to identification difficulties, pupal exuviae of *Chironomus* (s.str.) were not analysed further.

Our results confirmed the presence of previously doubtful records for Ukraine, such as *Diplocladius cultriger, Parorthocladius nudipennis, Synendotendipes dispar, Cladotanytarsus atridorsum, Paratanytarsus austriacus* and *Paratanytarsus lauterborni* (Spies and Sæther 2013). Ten species represent first records for the Ukrainian fauna.

## Checklists

### List of newly recorded Chironomidae species from Ukraine

#### Zavrelimyia melanura

(Meigen, 1804)

C4B14CD8-E6C0-51D2-B80D-345E764AA95D

##### Materials

- **Type status:** Other material. **Occurrence:** recordedBy: M. N.; individualCount: 4; lifeStage: pupal exuviae; occurrenceID: BDJ_13073_1; **Location:** country: Ukraine; locality: Svydovets, lake Geryshaska; verbatimElevation: 1584; **Event:** eventDate: 26-06-19

##### Distribution

Palaearctic species distributed in Europe, Far East, Near East and North Africa.

##### Notes

Larvae of Zavrelimyia are common components of littoral assemblages of mountain lakes in the Alps (Boggero et al. 2006, Lods-Crozet et al. 2012, Boggero 2018), South Carpathians (Tatole 2004) and the Tatra Mountains (Bitušík et al. 2006). The species is cold-stenothermic and, in addition to lakes, it occurs in mountain streams and rivers (e.g. Laville 1980, Casas and Vilchez-Quero 1993, Rossaro et al. 2006).

#### Acamptocladius reissi

Cranston et Saether, 1982

A6FC8F95-7A44-5441-A002-744A854B28D8

##### Materials

- **Type status:** Other material. **Occurrence:** recordedBy: M. N.; individualCount: 1; lifeStage: pupal exuviae; occurrenceID: BDJ_13073_2; **Location:** country: Ukraine; locality: Svydovets, lake Geryshaska; verbatimElevation: 1584; **Event:** eventDate: 26 -06-19

##### Distribution

Palaearctic species, sporadically distributed in nine European countries.

##### Notes

The species is known from mountain peat pools and peatland lakes (Baars et al. 2014); however Ferrarese and Lencioni (2003) found pupae and larvae in the littoral of an alpine lake of glacial origin at an altitude of 1936 m in Central-Eastern Alps. The lake in their study resembled our lake Geryshaska by the Carex-dominated littoral and general character of its surroundings.

#### Cricotopus (Isocladius) speciosus

Goetghebuer, 1921

56ABFDFF-6BC3-5832-B29E-CE46B486F5D6

##### Materials

- **Type status:** Other material. **Occurrence:** recordedBy: M. N.; individualCount: 12; lifeStage: pupal exuviae; occurrenceID: BDJ_13073_3; **Location:** country: Ukraine; locality: Svydovets, lake Vorozheska 1; verbatimElevation: 1480; **Event:** eventDate: 26-06-19

##### Distribution

Palaearctic species, recently known from seven West European countries, European part of Russia and from East Palaearctic.

##### Notes

Larvae of the subgenus Isocladius are widespread in mountain lakes in the Alps (Boggero 2018) and the Tatra Mountains (Bitušík et al. 2006), but species composition is insufficiently known due to identification difficulties. C. (I.) speciosus belongs to the *sylvestris* group, members of which are mostly eurythermic and euryoecious. There is a considerable gap in the knowledge of the species' ecologies. Langton and Visser (2003) mention its occurrence in ponds, lakes and running waters.

#### Cricotopus (Cricotopus) curtus

Hirvenoja 1973

93FFAF97-633A-5980-889A-2F5FF83C403F

##### Materials

- **Type status:** Other material. **Occurrence:** recordedBy: M. N; individualCount: 1; lifeStage: pupal exuviae; occurrenceID: BDJ_13073_4; **Location:** country: Ukraine; locality: Svydovets, lake Vorozheska 1; verbatimElevation: 1480; **Event:** eventDate: 26-06-19

##### Distribution

Holarctic species recorded in most countries in Western and Central Europe. Major gaps in distribution include the Balkans and a belt from Scandinavia to South-European Russia.

##### Notes

A common rheophilic and polyoxybiontic species. Pupal exuviae could originate both from the inlet and the littoral of the lake, as slow-flow conditions are present along the lake shores.

#### Heterotrissocladius marcidus

Walker, 1856

9E991ADD-BF7B-5339-AE98-E85FC2CD1A2D

##### Materials

- **Type status:** Other material. **Occurrence:** recordedBy: M. N.; individualCount: 1; lifeStage: pupal exuviae; occurrenceID: BDJ_13073_5; **Location:** country: Ukraine; locality: Chornohora, lake Nesamovyte; verbatimElevation: 1745; **Event:** eventDate: 24-06-19
- **Type status:** Other material. **Occurrence:** recordedBy: M. N.; individualCount: 2; lifeStage: pupal exuviae; occurrenceID: BDJ_13073_6; **Location:** country: Ukraine; locality: Chornohora, lake Dantsyzh; verbatimElevation: 1671; **Event:** eventDate: 24-06-19

##### Distribution

Holarctic species widespread in Europe with the exception of the Balkans and a belt extending from the Baltics to Ukraine.

##### Notes

Belongs to the most widespread and often most abundant species in lakes of the Alps (Boggero et al. 2006) and the Tatra Mountains (Bitušík et al. 2006). Cogălniceanu et al. (2009) reported it for several lakes in the Retezat Mts., Romania. We would expect its occurrence in lakes situated at higher altitudes in the Ukrainian part of the Eastern Carpathians.

#### Orthocladius (Orthocladius) dentifer

Brundin, 1947

E23648EF-9AB8-5774-B2EC-6511AF74E50C

##### Materials

- **Type status:** Other material. **Occurrence:** recordedBy: M. N.; individualCount: 2; lifeStage: pupal exuviae; occurrenceID: BDJ_13073_7; **Location:** country: Ukraine; locality: Svydovets, lake Geryshaska; verbatimElevation: 1584; **Event:** eventDate: 26-06-19
- **Type status:** Other material. **Occurrence:** recordedBy: M. N.; individualCount: 16; lifeStage: pupal exuviae; occurrenceID: BDJ_13073_8; **Location:** country: Ukraine; locality: Svydovets, lake Vorozheska 1; verbatimElevation: 1480; **Event:** eventDate: 26-06-19
- **Type status:** Other material. **Occurrence:** recordedBy: M. N.; individualCount: 76; lifeStage: pupal exuviae; occurrenceID: BDJ_13073_9; **Location:** country: Ukraine; locality: Svydovets, lake Vorozheska 2; verbatimElevation: 1477; **Event:** eventDate: 26-06-19
- **Type status:** Other material. **Occurrence:** recordedBy: M. N.; individualCount: 3; sex: M; lifeStage: pharate adult; occurrenceID: BDJ_13073_10; **Location:** country: Ukraine; locality: Svydovets, lake Vorozheska 2; verbatimElevation: 1477; **Event:** eventDate: 26-06-19

##### Distribution

Holarctic species, known from Western and Northern Europe but not previously recorded in Central and Eastern Europe.

##### Notes

Larvae of *Orthocladius* (s. l.) are rheophilic to rheobiontic and poloxybiontic, generally confined to well-aerated flowing waters. They are recorded in the littoral of alpine lakes (Boggero et al. 2006), mostly identified to genus/subgenus level. *Orthocladius dentifer* is known from lakes (Rossaro et al. 2003) and rivers (Murray et al. 2014), even in severely polluted conditions (Loskutova et al. 2015).

#### Psectrocladius (Psectrocladius) oligosetus

Wuelker, 1956

B3E7CF7D-A874-518C-B34F-B747395B06A5

##### Materials

- **Type status:** Other material. **Occurrence:** recordedBy: M. N.; individualCount: 4; lifeStage: pupal exuviae; occurrenceID: BDJ_13073_11; **Location:** country: Ukraine; locality: Chornohora, lake Breskul 1; verbatimElevation: 1738; **Event:** eventDate: 23-06-19
- **Type status:** Other material. **Occurrence:** recordedBy: M. N.; individualCount: 5; lifeStage: pupal exuviae; occurrenceID: BDJ_13073_12; **Location:** country: Ukraine; locality: Chornohora, lake Breskul 2; verbatimElevation: 1728; **Event:** eventDate: 23-06-19

##### Distribution

West Palaearctic species, in Europe known from western and northern countries, with distribution gaps from the Baltic republics across Poland and Ukraine to the Balkans.

##### Notes

Apparently a cold-stenothermic species occurring in lakes in mountain regions (e.g. Rieradevall et al. 2007, Bitušík and Svitok 2006, Bitušík et al. 2007, Boggero 2018).

#### Polypedilum (Pentapedilum) uncinatum

(Goetghebuer, 1921)

7ABC8E09-8DFB-5725-9839-5584A2DDF6A1

##### Materials

- **Type status:** Other material. **Occurrence:** recordedBy: M. N.; individualCount: 5; lifeStage: pupal exuviae; occurrenceID: BDJ_13073_13; **Location:** country: Ukraine; locality: Svydovets, lake Geryshaska; verbatimElevation: 1584; **Event:** eventDate: 26-06-19
- **Type status:** Other material. **Occurrence:** recordedBy: M. N.; individualCount: 1; lifeStage: pupal exuviae; occurrenceID: BDJ_13073_14; **Location:** country: Ukraine; locality: Svydovets, lake Vorozheska 2; verbatimElevation: 1477; **Event:** eventDate: 26-06-19

##### Distribution

Holarctic species recorded from a small number of European countries; however, its distribution from Scandinavia to Greece indicates its potential occurrence all over Europe.

##### Notes

The species belongs to typical chironomid generalists for temporary wetlands, with adaptation to survive dry periods in moist soil (Dettinger-Klemm 2003).

#### Paratanytarsus laccophilus

(Edwards, 1929)

982FE5E6-2D31-5618-A8AB-D78E3FE8934C

##### Materials

- **Type status:** Other material. **Occurrence:** recordedBy: M. N.; individualCount: 31; lifeStage: pupal exuviae; occurrenceID: BDJ_13073_15; **Location:** country: Ukraine; locality: Chornohora, lake Breskul 1; verbatimElevation: 1738; **Event:** eventDate: 23-06-19
- **Type status:** Other material. **Occurrence:** recordedBy: M. N.; individualCount: 23; lifeStage: pupal exuviae; occurrenceID: BDJ_13073_16; **Location:** country: Ukraine; locality: Chornohora, lake Nesamovyte; verbatimElevation: 1745; **Event:** eventDate: 24-06-19
- **Type status:** Other material. **Occurrence:** recordedBy: M. N.; individualCount: 155; lifeStage: pupal exuviae; occurrenceID: BDJ_13073_17; **Location:** country: Ukraine; locality: Svydovets, lake Geryshaska; verbatimElevation: 1584; **Event:** eventDate: 26-06-19
- **Type status:** Other material. **Occurrence:** recordedBy: M. N.; individualCount: 2; sex: M; lifeStage: pharate adult; occurrenceID: BDJ_13073_18; **Location:** country: Ukraine; locality: Svydovets, lake Geryshaska; verbatimElevation: 1584; **Event:** eventDate: 26-06-19
- **Type status:** Other material. **Occurrence:** recordedBy: M. N.; individualCount: 1; lifeStage: pupal exuviae; occurrenceID: BDJ_13073_19; **Location:** country: Ukraine; locality: Svydovets, lake Vorozheska 1; verbatimElevation: 1480; **Event:** eventDate: 26-06-19

##### Distribution

Holarctic species, primarily distributed in northern and Western Europe, records are lacking from the southern and eastern part of the continent.

##### Notes

Euryoecious species occurring in ponds and lakes (Langton and Visser 2003). It belongs to typical colonisers of both natural (Lencioni et al. 2018) and artificial water bodies (Bukvová and Hamerlík 2015). However, the species is not characteristic for high mountain lakes. While Boggero (2018) considered *P. laccophilus* to be rare in lakes of the southern side of the Central-Eastern Alps, it has not been found in the Tatra Mountains at all.

#### Tanytarsus bathophilus

Kieffer, 1911

2BB7061C-8F37-5770-878A-C99375BE4D19

##### Materials

- **Type status:** Other material. **Occurrence:** recordedBy: M. N.; individualCount: 12; lifeStage: pupal exuviae; occurrenceID: BDJ_13073_20; **Location:** country: Ukraine; locality: Svydovets, lake Vorozheska 1; verbatimElevation: 1480; **Event:** eventDate: 26-06-19
- **Type status:** Other material. **Occurrence:** recordedBy: M. N.; individualCount: 2; lifeStage: pupal exuviae; occurrenceID: BDJ_13073_21; **Location:** country: Ukraine; locality: Svydovets, lake Vorozheska 2; verbatimElevation: 1477; **Event:** eventDate: 26-06-19

##### Distribution

Palaearctic species widespread in Europe, but with a major gap in occurrence extending from the Baltic Republics to Southern Europe (apart from Romania) and the Balkans.

##### Notes

Larvae live mainly in lakes but also in flowing waters (e.g. Rossaro et al. 2006). It is the most commonly encountered species of the genus in alpine lakes in the Tatra Mountains (Bitušík et al. 2006), but apparently missing in alpine lakes of the South Carpathians (Tatole 2004, Cogălniceanu et al. 2009).

## Discussion

Our study contributes to the knowledge of the chironomid fauna of alpine lakes in the Ukrainian part of the Eastern Carpathians. We report a total of 34 species/taxa, while almost one third (10 species) of them were recorded for the first time in Ukraine. We are aware that this inventory is far from complete, as evidenced by the comparison with data from the Tatra Mountains (Western Carpathians) and the South Carpathians (Tatole 2004, Bitušík et al. 2006, Cogălniceanu et al. 2009).

The most common species of the Tatra Mts. lakes (Western Carpathians, Bitušík et al. 2006) and the Retezat Mts. (South Carpathians, Cogălniceanu et al. 2009), *Heterotrissocladius marcidus* and *Paratanytarsus austriacus* were found only in a few of the Ukrainian lakes. On the other hand, the most characteristic species of the surveyed lakes were either not present in the Tatra Mts. lakes (e.g. *Cladotanytarsus atridorsum* and *Paratanytarsus laccophilus*) or were very rare (e.g. *Chironomus* spp., *Synendotendipes* sp.) (Bitušík et al. 2006). Nevertheless, the last two taxa can be common in acidic Tatra Mts. ponds situated in lower altitudes (Novikmec et al. 2015, Hamerlík et al. 2016). Absence of the members of the Diamesinae subfamily, typical for cold, nutrient-poor alpine lakes, from the Ukrainian samples is also interesting; however, it does not necessarily mean that they are not present in the lakes; their absence was most likely caused by spring emergence of the adults (e.g. Raunio et al. 2010). In general, chironomid communities of Ukrainian alpine lakes represent a mixture of species typical for cold alpine lakes and acidic ponds situated in lower altitudes.

The results of this “snap-shot” survey are important for at least two reasons: 1) it is the first insight into species composition of chironomid assemblages of Ukrainian alpine lakes in the context of the whole Carpathians and 2) the data can be useful in determining the ecological conditions in the alpine lakes and can create a basis for future (paleo)limnological studies extended to the whole „alpine lake district“ in the Ukrainian Carpathians.

## Supplementary Material

XML Treatment for Zavrelimyia melanura

XML Treatment for Acamptocladius reissi

XML Treatment for Cricotopus (Isocladius) speciosus

XML Treatment for Cricotopus (Cricotopus) curtus

XML Treatment for Heterotrissocladius marcidus

XML Treatment for Orthocladius (Orthocladius) dentifer

XML Treatment for Psectrocladius (Psectrocladius) oligosetus

XML Treatment for Polypedilum (Pentapedilum) uncinatum

XML Treatment for Paratanytarsus laccophilus

XML Treatment for Tanytarsus bathophilus

## Figures and Tables

**Table 1.** Basic characteristics of the studied lakes. ^a^Tsarenko et al. (2019)

Lake name	Location	Altitude (m)	Max. depth (m)	Area (ha)
Nesamovyte	48.12238 N, 24.53945 E	1745	2.0^a^	0.35
Breskul 1	48.14943 N, 24.50369 E	1738	1.1	0.04
Breskul 2	48.14813 N, 24.50411 E	1728	1.6	0.01
Dantsyzh	48.13129 N, 24.53715 E	1671	0.9	0.05
Geryshaska	48.26978 N, 24.16531 E	1584	2.0	1.90
Vorozheska 1	48.27612 N, 24.19274 E	1480	2.2	0.54
Vorozheska 2	48.27746 N, 24.19270 E	1477	0.8	0.12
Vorozheska 3	48.27748 N, 24.19331 E	1469	0.3	0.01

**Table 2.** List of recorded chironomid species/taxa in the surveyed lakes. Numbers without symbol represent pupal exuviae, symbols # – new record for Ukraine, ⸸ – previously considered doubtful, * – adult male, ** – pharate adult (male). Abbreviations of lake names: Nes – Nesamovyte, Bre1 – Breskul 1, Bre2 – Breskul 2, Dan – Dantsyzh, Ger – Geryshaska, Vor1 – Vorozheska 1, Vor2 – Vorozheska, Vor3 – Vorozheska 3.

**Taxa**	**Lake name**							
	**Nes**	**Bre1**	**Bre2**	**Dan**	**Ger**	**Vor1**	**Vor2**	**Vor3**
Tanypodinae	-	-	-	-	-	-	-	-
Procladius (Holocladius) choreus (Meigen, 1804)	-	7, 1**	-	-	-	45, 1**	19, 1*	-
*Macropelopia nebulosa* Meigen, 1804	1	-	-	13	-	-	-	-
# *Zavrelimyia melanura* (Meigen, 1804)	-	-	-	-	-	4	-	-
Prodiamesinae	-	-	-	-	-	-	-	-
*Prodiamesa olivacea* (Meigen, 1818)	-	-	-	1	-	-	-	-
Orthocladiinae	-	-	-	-	-	-	-	-
# *Acamptocladius reissi* Cranston et Saether, 1982	-	-	-	-	1	-	-	-
Corynoneura cf. coronata Edwards, 1924	-	3	-	-	-	-	-	-
Corynoneura cf. fittkaui Schlee, 1968	2*	3, 3*	3	-	-	-	-	-
*Corynoneura* Pe2a Langton, 1991	1	-	-	-	18	-	-	-
# Cricotopus (Isocladius) speciosus Goetghebuer, 1921	-	-	-	-	-	12	-	-
Cricotopus (Isocladius) sylvestris Fabricius, 1794)	-	-	-	-	2, 1**	3	-	-
Cricotopus (Isocladius) Pe 5 Langton, 1991	-	-	-	-	1	-	-	-
Cricotopus (Isocladius) intersectus (Staeger, 1839)	44, 4*	-	-	-	-	-	-	-
# Cricotopus (Cricotopus) curtus Hirvenoja 1973	-	-	-	-	-	1	-	-
⸸ *Diplocladius cultriger* Kieffer, 1908	-	-	-	4, 1**	-	-	-	-
# *Heterotrissocladius marcidus* Walker, 1856	1	-	-	2	-	-	-	-
Nanocladius (Nanocladius) parvulus (Kieffer, 1909)	-	-	-	-	-	-	2	-
# Orthocladius (Orthocladius) dentifer Brundin, 1947	-	-	-	-	2	16	76, 3**	-
⸸ *Parorthocladius nudipennis* (Kieffer, 1908)	-	-	-	-	-	1	-	-
# Psectrocladius (Psectrocladius) oligosetus Wuelker, 1956	-	4	5	-	-	-	-	-
Chironominae - Chironomini	-	-	-	-	-	-	-	-
*Benthalia carbonaria* (Meigen, 1804)	-	-	-	-	40	-	-	-
Chironomus (Chironomus) spp.	6	9	-	-	9	25	19	21
*Chironomus* lob-pe 2a Langton & Visser 2003	-	16	33	-	-	-	-	5
*Pagastiella orophila* (Edwards, 1929)	40, 3*	-	-	-	-	-	-	-
# Polypedilum (Pentapedilum) uncinatum (Goetghebuer, 1921)	-	-	-	-	5, 10*	-	1	-
genus *Synendotendipes* Langton et Visser, 2003	1	14	9	-	2	-	-	5
⸸ *Synendotendipes dispar* (Meigen, 1830)	-	-	-	-	-	-	-	2*
Chironominae - Tanytarsini	-	-	-	-	-	-	-	-
⸸ Cladotanytarsus (Cladotanytarsus) atridorsum Kieffer, 1924	-	-	-	-	50	103, 6*	50, 1*	1
*Micropsectra lindrothi* Goetghebuer, 1931	-	-	-	5	-	-	-	-
⸸ *Paratanytarsus austriacus* (Kieffer, 1924)	-	-	-	-	-	14	10	-
# *Paratanytarsus laccophilus* (Edwards, 1929)	23	31	-	-	155, 2*	1	-	-
⸸ *Paratanytarsus lauterborni* (Kieffer, 1909)	-	-	-	-	-	2	12	-
# *Tanytarsus bathophilus* Kieffer, 1911	-	-	-	-	-	12	2	-
*Tanytarsus gregarius* Kieffer, 1909	93, 4*	-	-	-	-	-	-	-
*Tanytarsus* Pe 4c Langton, 1991	-	-	-	-	6	-	-	-
**No of taxa**	**10**	**8**	**4**	**5**	**12**	**13**	**9**	**5**
